# Synergistic advantages of combining a traction device with underwater conditions for colonic endoscopic submucosal dissection

**DOI:** 10.1055/a-2598-5291

**Published:** 2025-06-26

**Authors:** Naoya Tada, Naoto Tamai, Mamoru Ito, Toshiki Futakuchi, Masakuni Kobayashi, Akira Dobashi, Kazuki Sumiyama

**Affiliations:** 1 12839 Department of Endoscopy, The Jikei University School of Medicine, Tokyo, Japan

Several reports have recently highlighted the efficacy of endoscopic submucosal dissection (ESD) in underwater conditions 1 2 3 . However, in underwater conditions alone, the traction force remains insufficient because a part of the resected specimen floats freely. To address this, we describe a novel technique comprising a traction device (Multi-loop traction device [MLTD]; Boston Scientific Japan, Tokyo, Japan) ( Fig. 1 ) 4 5 and underwater conditions (distilled water) for ESD of a large colorectal neoplasm ( Video 1 ).

**Fig. 1:**
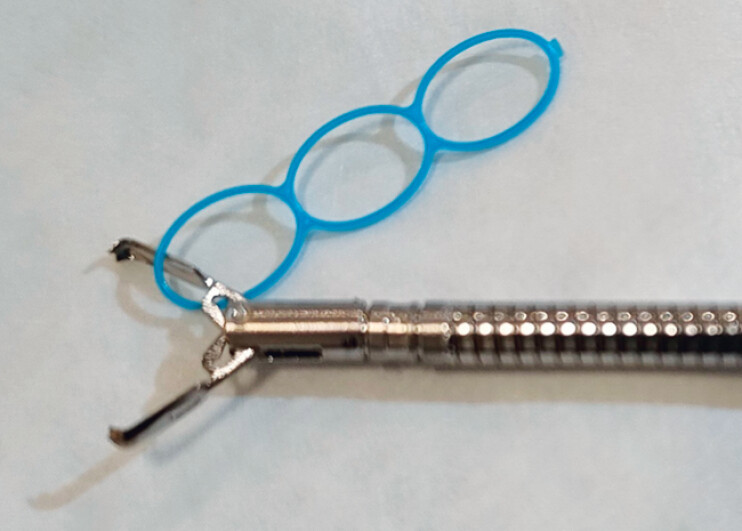
The Multi-loop traction device (MLTD; Boston Scientific Japan, Tokyo, Japan) with a reopenable clip (SureClip 8 mm; Micro-Tech, Nanjing, China). The MLTD consists of three interconnected loops, each 6–7 mm in size, made from linear low-density polyethylene.

Novel technique combining a traction device and underwater conditions for endoscopic submucosal dissection of a large colonic neoplasm.Video 1

A 61-year-old man was referred to our hospital for the endoscopic resection of a 45-mm neoplasm in the transverse colon. Based on Kudo’s pit pattern classification, the lesion exhibited a V _I_ pit pattern with mild irregularity, suggesting high grade dysplasia (intramucosal carcinoma), prompting a decision to perform ESD to achieve en bloc resection ( Fig. 2 ).

**Fig. 2:**
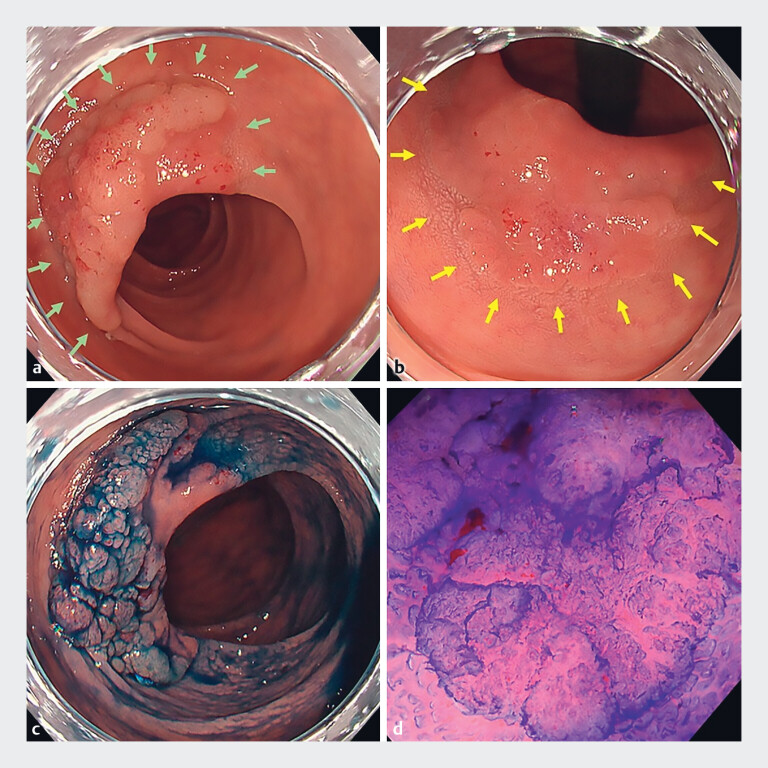
Imaging of the lesion. **a** Anal side of the large neoplasm in the transverse colon (green arrows). **b** Oral side of the colonic neoplasm (yellow arrows). **c** Anal side of the lesion with indigo carmine staining. **d** Crystal violet dye-magnifying endoscopy showing V _I_ pit pattern with mild irregularity based on Kudo’s pit pattern classification.

After the submucosal injection of hyaluronic acid, a circumferential incision was made under underwater conditions. The MLTD was fixed on the anal side of the lesion and the opposite side of the intestinal wall with reopenable clips to expose the submucosa ( Fig. 3 **a** ). The combined effect of traction-induced tension and the floating force in the water provided a clear view of the submucosa from the right edge to the left edge ( Fig. 3 **b, c** ). This enabled precise recognition of the cutting line, facilitating safe and effective submucosal dissection. Additionally, traction with an MLTD not only exposed the submucosa but also increased its tension, further optimizing the efficiency of dissection. When minor bleeding occurred, the bleeding site was easily identified, and hemostasis was quickly achieved in the water ( Fig. 4 ). En bloc resection was successfully completed in 56 minutes without any adverse events ( Fig. 5 ).

**Fig. 3:**
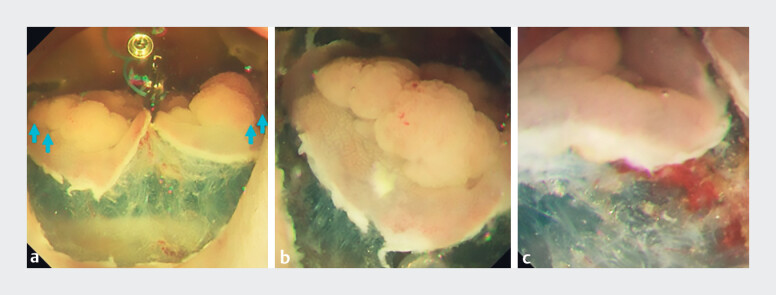
Endoscopic images. **a** The Multi-loop traction device (Boston Scientific Japan, Tokyo, Japan) was attached to the center of the lesion and the opposite side of the gastrointestinal wall, exposing the extensive submucosa under underwater conditions. Floating forces in underwater conditions also strengthened the traction forces of both edges of the lesion (blue arrows). **b** The left edge of the lesion. **c** The right edge of the lesion.

**Fig. 4:**
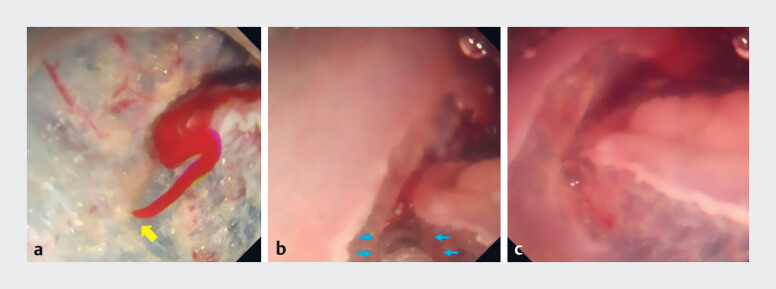
Endoscopic images. **a** The bleeding site was easily identified under underwater conditions (yellow arrow). **b** Hemostasis was achieved quickly by coagulation with a needle-knife (blue arrows). **c** After hemostasis.

**Fig. 5:**
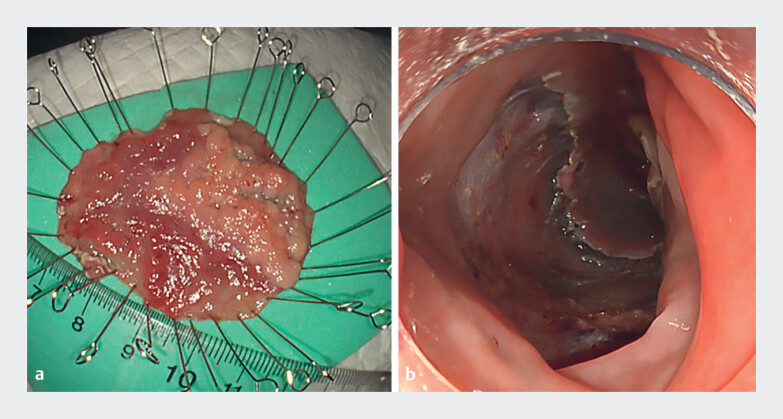
The resected specimen and mucosal defect. **a** The resected specimen (50 × 43 mm). Histopathological examination showed intramucosal carcinoma with negative horizontal and vertical resection margins. **b** The large defect occupied more than half of the intestinal lumen.

Colonic ESD with a traction device under underwater conditions might be an easy and effective technique for further enhancing the advantages of underwater conditions, thereby minimizing the influence of gravity and reducing procedural difficulty.

Endoscopy_UCTN_Code_TTT_1AQ_2AD

## Notes

Endoscopy E-Videos https://eref.thieme.de/e-videos
*E-Videos* is an open access online section of the journal *Endoscopy* , reporting on interesting cases and new techniques in gastroenterological endoscopy. All papers include a high-quality video and are published with a Creative Commons CC-BY license. Endoscopy E-Videos qualify for HINARI discounts and waivers and eligibility is automatically checked during the submission process. We grant 100% waivers to articles whose corresponding authors are based in Group A countries and 50% waivers to those who are based in Group B countries as classified by Research4Life (see: https://www.research4life.org/access/eligibility/ ). This section has its own submission website at https://mc.manuscriptcentral.com/e-videos .
